# Out-of-Pocket Expenditure on Chronic Non-Communicable Diseases in Sub-Saharan Africa: The Case of Rural Malawi

**DOI:** 10.1371/journal.pone.0116897

**Published:** 2015-01-13

**Authors:** Qun Wang, Alex Z. Fu, Stephan Brenner, Olivier Kalmus, Hastings Thomas Banda, Manuela De Allegri

**Affiliations:** 1 Institute of Public Health, Faculty of Medicine, University of Heidelberg, Heidelberg, Germany; 2 Cancer Prevention and Control Program, Georgetown University Medical Center, Washington, D.C., United States of America; 3 Research for Equity and Community Health Trust (REACH Trust), Lilongwe, Malawi; University of Milan, ITALY

## Abstract

In Sub-Saharan Africa (SSA) the disease burden of chronic non-communicable diseases (CNCDs) is rising considerably. Given weaknesses in existing financial arrangements across SSA, expenditure on CNCDs is often borne directly by patients through out-of-pocket (OOP) payments. This study explored patterns and determinants of OOP expenditure on CNCDs in Malawi. We used data from the first round of a longitudinal household health survey conducted in 2012 on a sample of 1199 households in three rural districts in Malawi. We used a two-part model to analyze determinants of OOP expenditure on CNCDs. 475 respondents reported at least one CNCD. More than 60% of the 298 individuals who reported seeking care incurred OOP expenditure. The amount of OOP expenditure on CNCDs comprised 22% of their monthly per capita household expenditure. The poorer the household, the higher proportion of their monthly per capita household expenditure was spent on CNCDs. Higher severity of disease was significantly associated with an increased likelihood of incurring OOP expenditure. Use of formal care was negatively associated with the possibility of incurring OOP expenditure. The following factors were positively associated with the amount of OOP expenditure: being female, Alomwe and household head, longer duration of disease, CNCDs targeted through active screening programs, higher socio-economic status, household head being literate, using formal care, and fewer household members living with a CNCD within a household. Our study showed that, in spite of a context where care for CNCDs should in principle be available free of charge at point of use, OOP payments impose a considerable financial burden on rural households, especially among the poorest. This suggests the existence of important gaps in financial protection in the current coverage policy.

## Introduction

The World Health Organization (WHO) describes burden of disease into three groups: communicable and maternal and perinatal diseases; non-communicable diseases; and injuries [1]. In this study we focused on non-communicable diseases that affect people for a prolonged time and used the terminology of chronic non-communicable diseases (CNCDs) to represent these conditions [2]. Globally, CNCDs account for the greatest share of premature deaths and disabilities [3,4]. Beyond the disease burden, CNCDs also pose a substantial financial burden [4]. The treatment of clinically manifested CNCDs is assumed to be less cost-effective than early modification and prevention of CNCD risk factors [5]. In low- and middle-income countries (LMICs) where at least 80% of all worldwide deaths related to CNCDs occur [3,4], the economic burden of CNCDs is especially high [4–6].

In Sub-Saharan Africa (SSA), the disease burden of CNCDs is rising considerably, with the continent being predicted to witness the greatest worldwide increase of deaths attributable to CNCDs [7]. This rapid increase of CNCDs in SSA represents an important burden to national health care systems as they are already strained in their capacity to provide full coverage of quality maternal and communicable disease services and to ensure adequate financial protection for their populations [8,9]. In spite of already living at the margin of poverty, the additional financial burden of CNCD healthcare costs is further shifted to local populations, who are expected to contribute through direct out-of-pocket (OOP) spending [10,11].

Given this increasing disease burden, both understanding the patterns of OOP spending as well as identifying sub-groups at specific risk of incurring high OOP spending for CNCDs represent essential steps towards the development of adequate policies that protect those most at risk from facing impoverishment due to chronic illness. However, very few pertinent studies have been conducted from an economic viewpoint, with most of them primarily aimed at only estimating the overall financial burden imposed by CNCDs to local populations [11–18]. To our knowledge, no study has been conducted so far to specifically identify those factors associated with OOP expenditure on CNCDs in SSA. While some studies on expenditure analysis in SSA have included CNCDs as an explanatory variable influencing the level of OOP expenditure on healthcare, they failed to either specifically describe the patterns and determinants of OOP spending on CNCDs [19,20], or to clearly differentiate acute from chronic conditions [15,21]. Our population-based study therefore aimed at filling this knowledge gap by describing patterns of household OOP spending on CNCDs and exploring their determinants in the context of rural Malawi. The objective in this study is to identify which factors place people at risk of incurring OOP expenditure and specifically higher OOP expenditure on CNCDs.

## Methods

### Study setting

Malawi is a low-income country in SSA. CNCDs increasingly contribute to the disease burden to be shouldered by Malawians [22]. In 2008, CNCDs were estimated to account for 28% of all deaths in Malawi, with cardiovascular diseases, cancers, chronic respiratory diseases, and diabetes being the leading causes of mortality [23].

The country’s health financing structure relies on general tax revenue and external donor funds. Officially, all health services provided through the formal public sector are supposed to be free of charge. To further guide national policy makers and international development agencies, the Ministry of Health (MoH) in 2004 defined a specific Essential Health Package (EHP) outlining a minimum package of cost-effective health interventions. The EHP is provided free of charge at all public health facilities and at selected non-profit private facilities contracted by the MoH through service level agreements. Initially, the EHP covered only the costs related to prevention and treatment of communicable, maternal, perinatal, and nutritional conditions. In 2010, the EHP was expanded to include coverage for common CNCDs, such as the screening, prevention, and treatment of cardiovascular, diabetic, and certain cancerous diseases (breast cancer, cervical cancer), therapeutic approaches targeting forms of chronic mental illness, and the general promotion of healthy life-styles in relation to CNCDs [24,25]. Prior work by the authors indicated that a substantial portion of people suffering from CNCDs do not seek any care even for chronic conditions which are, in principle, covered by the EHP [26].

Our study was conducted in Thyolo, Chiradzulu, and Mulanje, three rural districts in Southern Malawi. Approximately 1.4 million people live in these three districts, equivalent to 11% of the country population [27]. At the time of the study, these three districts counted a total of 60 public health facilities, 16 private non-profit health facilities operated by the Christian Health Association of Malawi, and 6 private for-profit health facilities.

### Data and data collection

We obtained ethical approval from Ethikkommission der Medizinischen Fakultät Heidelberg (the ethical committee of the faculty of Medicine, Heidelberg University, Germany) and the National Health Sciences Research Committee, Ministry of Health, Malawi. Written consent was obtained from each respondent in their primary language. We used data from the first wave of a longitudinal household health survey conducted in these three districts from August to October 2012 on a representative sample of 1199 households spread across 77 villages using a two-stage sampling procedure. Sampling strategies, informed by the long-term wish to evaluate the impact of an upcoming micro-health insurance scheme planned for implementation in the region, are described in detail elsewhere [26]. Data was collected using a fully digitalized system by means of tablet computers and direct data transfer over mobile phone networks. The questionnaire was administered face to face to each member of a household and gathered information on a household’s socio-demographic and economic profile as well as household members’ individual illness profiles on both acute and chronic conditions.

To ensure completeness of self-reported information on CNCDs, we collected illness information on both medical terminology (e.g. chronic disorders based on clinical diagnoses or clinical symptoms provided to individuals) and lay person phrasing (commonly used descriptions of chronic impairment or disabilities) derived from the WHO’s International Classification [28]. In consonance with previous studies, we defined chronic diseases as any illness or complaint that lasted longer than three months or any illness or complaint that occurred earlier in a respondent’s life and continued to affect the individual’s health status at the time of the interview [12]. In cases with multiple reported illnesses or complaints, interviewers were instructed to code them as primary and secondary diagnoses or symptoms based on respondents’ descriptions. The obtained information on chronic diagnoses and symptoms was further categorized into one of ten non-communicable illness categories based on the WHO categories used for reporting the global disease burden of non-communicable diseases [1].

Once respondents reported one or more chronic illnesses or complaints, the reviewers continued to ask a series of additional questions on utilization of health care services and OOP expenditure related to these chronic conditions. In line with prior research [15,19,21,29,30], information on health service utilization and expenditure was limited to a four-week recall period.

### Variables and their measurement

Table 1 presents all variables included in this study and their measurement. We defined the primary outcome as an individual’s four-week OOP expenditure on CNCDs, including expenditure on medical care and related transport. OOP medical expenditure included consultation fees, and costs for laboratory tests, drugs, medical aids and disposables. Transport expenditure included only direct spending on transportation, but excluded the opportunity cost related to seeking care. The outcome variable was defined at the individual level. To counter the effect of extreme cases of expenditure data, the descriptive analysis of OOP expenditure relied on trimmed means. By excluding the smallest 5% and largest 5% of all cases in a sample, this approach allows for a better measurement of central tendency [31].

**Table 1:** Variables and measurements.

**Variable**	**Measurement**
Individual four-week OOP expenditure on CNCDs, including medical and transport spending (MWK)	Continuous variable
Age (years)	Continuous variable
Female	0 = No; 1 = Yes
Alomwe	0 = Other; 1 = Alomwe
Being household head	0 = No; 1 = Yes
Duration of CNCDs (years)	Continuous variable
Perceiving CNCD as serious	0 = No; 1 = Yes
CNCDs targeted by screening program	0 = No; 1 = Yes
Four-week per capita household expenditure (in 1000 MWK)	Used as continuous variable in two part model; Categorized into quartiles (1 = lowest SES; 4 = highest SES) for descriptive analysis
Household head being literate	0 = No; 1 = Yes
Proportion of people with CNCDs within the household	Continuous variable
Use of formal care	0 = No; 1 = Yes
Distance to nearest health facility (km)	Continuous variable

CNCD = chronic non-communicable diseases; OOP = out-of-pocket; MWK = Malawian Kwacha.

We included individual, household, and health system characteristics as explanatory variables. Individual level characteristics included: age, sex, ethnicity, being household head or not, illness duration, perceived illness severity, and the class of reported CNCD in relation to whether a specific risk factor screening program is offered by EHP or not. Ethnicity was defined as a dichotomous variable based on the major ethnic group (Alomwe) in the study area. Perceived severity was also defined as a dichotomous variable based on whether respondents perceived CNCDs to hinder themselves from conducting daily activities or keeping up desired life-styles. We further classified the ten WHO categories on CNCDs into two groups based on the specificities of the Malawian health system context: CNCD categories targeted by a risk factor screening program currently offered through the EHP; and CNCD categories not targeted by any EHP screening program, as described in detail in prior work [26] with the exception that individuals reporting chronic complaints related to cancerous diseases were further differentiated: Respondents reporting diagnoses or symptoms related to gynecological malignancies were included into the EHP-targeted CNCD group, all other respondents reporting cancers were included in the other group. The EPH-targeted CNCD group included cardiovascular diseases, diabetes mellitus, and gynecological malignancies (i.e. breast and cervical cancer), since these conditions are actively targeted by the EHP through early detection of hypertension, hyperglycemia, and precancerous/early stage tissue alterations. The group of non-EPH-targeted CNCDs included chronic pain syndromes, chronic respiratory syndromes, chronic problems with ears, nose, throat, or eyes, chronic mental health problems, chronic gastrointestinal syndromes, chronic skin problems, chronic genitourinary syndromes, other chronic problems, and non-gynecological cancers. The rationale for this division rested on the hypothesis that expenditure incurred may differ across the two condition groups, given that the former CNCD group can be targeted prior to the development of symptomatic manifestations while the latter cannot.

Household level factors included socio-economic status (SES), literacy of the household head, and the proportion of people with CNCDs living in a household. In line with prior studies [12,15,30,32], the four-week per capita household expenditure was used as a proxy for SES. This measurement included household expenditure on various items (e.g. food, alcohol, clothing, housing, transportation, communication, entertainment, education, personal care, insurance, transfers and remittances) over a four-week recall period. To ensure independence from the outcome variable, we excluded health expenditure from this computation. To adjust for differences across households of different sizes, we computed per capita household expenditure by dividing aggregated household expenditure by the number of people living within a household.

All expenditure information, whether related to the outcome or to the explanatory variables, is expressed in the local currency, the Malawian Kwacha (MWK), with the measurement unit set at 1 MWK. At the time of data collection, 1 US dollar was equivalent to 280 MWK. To facilitate data analysis, in the empirical model, we divided the four-week per capita household expenditure by 1,000 using ‘1,000 MWK’ as an adjusted unit [33].

Health system characteristics included respondents’ use of formal care and households’ distance to the nearest health facility. Use of formal care was defined as having sought care at a public facility, at a non-profit private facility, or at a private for-profit facility. Use of informal care was defined as having sought any form of traditional treatment or home treatment without consulting any formal care provider. Distance was computed using the GPS coordinates of the household and of the closest official referral healthcare facility.

### Analytical frameworks to model healthcare expenditure

Two different approaches are commonly used to model determinants of healthcare expenditure: single-equation modeling and multi-part modeling (including two-part models). Ordinary least square and Box-cox transformation both present a series of limitation in dealing with right-skewed distributions and a mass of observations at zero values, two intrinsic features of expenditure data [34–36], while generalized linear models (GLM) can effectively accommodates skewness and heteroscedasticity through variance-weighting [37]. Still, all these models are single-equation models and thus, they assume a single relationship between expenditure and explanatory variables [34]. Two-part models allow that different parts of the expenditure data to have various responses to explanatory variables. They model separately the possibility of incurring any expenditure from the magnitude of the expenditure in cases when at least some expenditure is incurred. This approach fits well the distribution of healthcare expenditure data with massive amounts of zero values [38].

The OOP expenditure data used in our study contained a massive amount of zero expenditure observations. Thus, we chose the two-part model to analyze our data. In addition, our positive OOP expenditure data were right-skewed and heteroscedastic. To counter these effect, we gave priority to the GLM for the second-part (expenditure level) of our two-part model, which has already been used in the literature in different contexts [39,40].

### Statistical analysis

We used descriptive statistics (mean and standard deviation) to outline the characteristics of the individuals in our sample who reported at least one CNCD and to compare respondents who incurred OOP expenditure to respondents who did not. We used the Spearman rank correlation test to explore the bivariate relationship between positive OOP expenditure and continuous explanatory variables (e.g. age, disease duration) and the Wilcoxon rank-sum test to explore differences in positive OOP expenditure in subgroups defined by categorical variables (e.g. sex, ethnicity, etc.).

To construct our two-part model, we used data from all those who sought health care (i.e. excluding those who did not seek care) without relying on trimmed data as we did for the descriptive analysis since GLM allows for skewness and heterogenerity. The first part, which relies on logistic regression to estimate the probability of incurred positive OOP expenditure among all users of health care, can be expressed as:

$$\ln[\frac{\mathrm{Prob}(y>0|x)}{1-\mathrm{Prob}(y>0|x)}]=\alpha +\sum \beta_{i}x_{i}$$

where y represents the outcome variable OOP expenditure on CNCDs, x_i_ represents a set of explanatory variables, β_i_ denotes coefficients of the corresponding estimates, α is the constant term.

For the second part of the model, the key to using GLM is whether researchers can identify an appropriate link (the transformation of expectation of the outcome variable) and family (a distribution that reflects the mean-variance relationship) functions [41]. We used Box-Cox test to specify link function [42] and the modified Park test to family function [43,44]. Through these tests, we found that our data showed a log transformation and an inverse Gaussian distribution. So it is appropriate to use GLM as the second-part of the model, which estimates the determinants of the amount of OOP expenditure, can be expressed as:

$$\ln(E(y|x))=\alpha +\sum \beta_{i}x_{i}$$

where E(y) represents the expected value of the outcome variable OOP expenditure. Other variables share similar notations with the first-part model.

We computed the model using the newly developed Stata command of tpm [45]. Consistent with a prior study [46], we also estimated income elasticity of healthcare expenditure, defined as the percentage change in healthcare expenditure relative to the percentage change in income [47,48], from the second part (expenditure level) model using the Stata command of margins.

In line with prior studies [30,49], we calculated the OOP expenditure intensity ratio by dividing the individual four-week OOP expenditure on CNCDs by the four-week per capita household expenditure. Due to the skewness of this ratio, we used trimmed means to measure its central tendency and the Kruskal-Wallis test to analyze its distribution across SES quartiles.

## Results

475 (8.4%) individuals out of a total sample of 5643 reported at least one CNCD. 298 (62.7%) of them sought some form of care, either formal or informal. 182 out of 202 (90.1%) of those seeking formal care chose public facilities. 196 out of 298 (65.8%) of those seeking care incurred some level of CNCD-related OOP expenditure in the four weeks prior to the survey date, while 102 (34.2%) did not. Among 196 of those incurring OOP expenditure on CNCDs, mean OOP expenditure was 981.13 MWK and 578.16 MWK for those using formal care and informal care respectively (Table 2). Out of the 196 individuals who incurred OOP expenditure, 123 (62.8%) only incurred spending on medical care, not on transport.

**Table 2:** Study samples and their characteristics.

	**CNCDs sample^a^ (N = 475)**		**Non-users^a^ (n = 177)**		**Users not incurring OOP expenditure (N = 102)**		**Users incurring OOP expenditure (N = 196)**		**Spearman R^b^**	**p-value^d^**
			
	**Mean**	**SD**	**Mean**	**SD**	**Mean**	**SD**	**Mean**	**SD**		
Individual OOP expenditure (MWK)^b^	-	-	-	-	-	-	755.76	1000.12	-	-
Age (years)	33.24	23.45	30.64	22.51	34.06	22.87	35.15	24.46	0.0945	0.1877
Duration of diseases (years)	8.70	9.98	9.42	10.20	7.25	8.09	8.81	10.61	0.0868	0.2266
Four-week per capita household expenditure (in 1000 MWK)	7.99	23.22	6.19	8.84	8.64	28.33	9.27	28.63	0.2359	0.0009
Proportion of people with CNCDs within the household	0.38	0.23	0.39	0.23	0.35	0.19	0.39	0.25	−0.0492	0.4939
Distance to nearest health facility (km)	2.26	1.31	2.26	1.28	2.32	1.23	2.22	1.38	−0.0233	0.7462
	N	%	N	%	N	%	N	%	Mean (MWK)^c^	p-value^e^
Female										
Yes	269	56.6	107	60.5	62	60.8	100	51.0	671.61	
No	206	43.4	70	39.5	40	39.2	96	49.0	875.91	0.8106
Ethnicity										
Alomwe	295	62.1	107	60.5	67	65.7	121	61.7	933.62	
Other	180	37.9	70	39.5	35	34.3	75	38.3	524.06	0.2017
Being household head										
Yes	147	31.0	39	22.0	35	34.3	73	37.2	788.66	
No	328	69.0	138	78.0	67	65.7	123	62.8	735.81	0.1247
Perceiving a CNCD as serious										
Yes	281	59.2	73	41.2	69	67.7	139	70.9	900.83	
No	194	40.8	104	58.8	33	32.4	57	29.1	587.92	0.1662
CNCDs targeted by EPH screening program										
Yes	80	16.8	29	16.4	22	21.6	29	14.8	2379.26	
No	395	83.2	148	83.6	80	78.4	167	85.2	697.25	0.2498
Household head literacy										
Yes	381	80.2	142	80.2	84	82.4	155	79.1	826.49	
No	94	19.8	35	19.8	18	17.7	41	20.9	566.49	0.5341
Use of formal care										
Yes	-	-	-	-	90	88.2	112	57.1	981.13	
No	-	-	-	-	12	11.8	84	42.9	578.16	0.0861

SD = standard deviation.

^a^ The characteristics of those reporting CNCDs and non-users are also presented by our previous paper [25].

^b^ Spearman rank correlation was used to explore the relationship between positive OOP expenditure and explanatory variables.

^c^ Trimmed mean was used to depict OOP expenditure for those incurring OOP expenditure and different subgroups out of those incurring OOP expenditure. The use of trimmed mean implied losing 18 out of 196 observations for both those incurring OOP expenditure and subgroups out of those incurring OOP expenditure.

^d^ P-value is for Spearman rank correlation.

^e^ P-value is for Wilcoxon rank-sum test, which was used to initially test the difference of positive OOP expenditure in subgroups defined by categorical variables (sex, ethnicity, etc.) based on 196 observations of those incurring OOP expenditure.

Table 2 presents the characteristics of the CNCDs sample as well as the two subgroups of health care users based on whether they incurred OOP expenditure or not. Compared to those who did not incur any OOP expenditure, individuals who incurred OOP expenditure were less likely to use formal care and suffer from CNCDs targeted by the EHP and more likely to be male. Four-week per capita household expenditure was positively correlated with OOP expenditure on CNCDs (p<0.05). Use of formal care was positively associated with positive OOP expenditure on CNCDs (p<0.1).

Table 3 shows the results of the two-part model. In the first-part logistic regression model, higher perceived disease severity was significantly associated with an increased likelihood of incurring OOP expenditure. Use of formal care was found to be negatively associated with the possibility of incurring OOP expenditure. In the second-part GLM with log link, we found that being female, Alomwe, household head, having a longer duration of disease, suffering from a CNCD targeted by EHP screening, higher SES, household head literacy, and use of formal care were all positively associated with the amount of OOP expenditure. The proportion of people with CNCDs living in a household was negatively associated with the amount of OOP expenditure.

**Table 3:** Two part model for determinants of OOP expenditure on CNCDs (first part: logit model, second part: generalized linear model with log link and inverse Gaussian distribution).

	**First part: probability of positive OOP expenditure**			**Second part: determinants of the amount of OOP expenditure**		
		
	**OR**	**95% CI**	**p-value**	**Coeff**	**95% CI**	**p-value**
Age	1.003	0.988, 1.018	0.673	0.004	−0.015, 0.023	0.682
Female	0.625	0.331, 1.181	0.148	1.362***	0.589, 2.135	0.001
Alomwe	0.864	0.495, 1.507	0.607	0.966**	0.310, 1.623	0.004
Being household head	0.869	0.412, 1.832	0.712	1.706**	0.589, 2.822	0.003
Duration of CNCDs	1.010	0.980, 1.042	0.513	0.028*	0.006, 0.049	0.013
Perceiving CNCD as serious	1.936*	1.039, 3.608	0.038	0.773	−0.150, 1.696	0.101
CNCDs targeted by EHP screening program	0.877	0.428, 1.800	0.721	0.952*	0.153, 1.751	0.019
Four-week per capita household expenditure	1.000	0.989, 1.010	0.928	0.004*	0.0001, 0.008	0.043
Household head being literate	0.813	0.411, 1.610	0.553	0.895*	0.062, 1.728	0.035
Proportion of people with CNCDs within the household	2.326	0.669, 8.094	0.184	−3.183**	−5.105, −1.261	0.001
Use of formal care	0.154***	0.077, 0.305	<0.001	1.031***	0.429, 1.634	0.001
Distance to nearest health facility	0.925	0.756, 1.130	0.444	−0.067	−0.391, 0.257	0.686

OR = odds ratio; CI = confidence interval.

* p< 0.05

** p<0.01

*** p<0.001.

Income elasticity, estimated from the second-part GLM, at the mean and median of four-week per capita household expenditure was calculated as 0.0494 and 0.0233 respectively. Average income elasticity for the 196 observations of those incurring OOP expenditure on CNCDs was 0.0475.

Table 4 shows the distribution of the OOP expenditure intensity ratio across SES quartiles. Those who incurred OOP expenditure spent, on average, the equivalent of 22.0% of their monthly per capita household expenditure on care related to CNCDs. This proportion differed significantly across SES quartiles, with the poorest households spending the highest proportion on care for CNCDs.

**Table 4:** The OOP expenditure intensity ratio (ratio between OOP expenditure on CNCDs and four-week per capita household expenditure).

	**Mean (SD)**	**Median**	**N**
1^st^ Quartile (lowest SES)	0.542^a^ (0.750)	0.303	42
2^nd^ Quartile	0.300^a^ (0.488)	0.186	21
3^rd^ Quartile	0.201^a^ (0.272)	0.092	58
4^th^ Quartile (highest SES)	0.113^a^ (0.139)	0.052	75
Total	0.220^a^ (0.309)	0.098	196
Kruskal-Wallis	χ^2^ = 11.999	p = 0.007	

^a^ The use of trimmed means implied losing 4 out of 42, 2 out of 21, 4 out of 58, 6 out of 75 and 18 out of 196 individuals for households in 1^st^, 2^nd^, 3^rd^, 4^th^ quartile and all those who incurred expenditure respectively.

## Discussion

To our knowledge, this study is one of the first studies exploring determinants of OOP expenditure on CNCDs in SSA. In line with prior studies [50,51], our findings confirm that a considerable portion of people suffering from a CNCD face relatively high OOP expenditure, in spite of a system which, in principle, should offer essential care free of charge at point of use. The OOP expenditure intensity ratio identified in this study is consistent with previous studies on financial burden imposed by CNCDs in other settings in SSA [11–16]. More importantly, our two-part model allowed us to identify various factors that shape an individual’s risk of incurring higher OOP expenditure when chronically ill. Those determinants show similarities as well as differences with the determinants found to drive OOP expenditure on non-chronic conditions [19,21,30,32,46,52]. Because studies on determinants of OOP expenditure, whether for chronic or for acute conditions, are very limited in SSA, we have enlarged the scope of our discussion from SSA to LMICs.

This study used a two-part model to explore determinants of OOP expenditure on CNCDs. As explained in the methods, we limited the analysis to those who used health care and exclude *a priori* the ones who did not use any care. Findings indicating what socio-demographic and economic characteristics differentiate users versus non-users among individuals reporting CNCDs are reported in previously published work [26]. We included users of health services without any OOP expenditure in the zero OOP expenditure group. This is based on the context of our study setting and our study perspective with a clear focus on individual OOP expenditure on CNCDs instead of the societal expenditure related to CNCDs. It is worth mentioning that individuals who used health care services related to CNCDs without incurring OOP expenditures are assumed to have consumed state-funded resources.

In line with prior studies [19,32,52,53], our findings revealed that the wealthier the household, the higher the absolute OOP expenditure on CNCDs. In our study, however, the role played by SES in shaping absolute OOP expenditure was less prominent than usually found in the literature [19,32,52,53]. Similarly, as shown previously, the role of SES in this Malawian setting was also less prominent in influencing health seeking behavior for CNCDs [26], which might indicate that SES in this context might be less important in explaining the differences in absolute OOP expenditure. In a system which, at least in principle provides care free of charge at point of use, absolute differences in OOP expenditure across SES quartiles are likely to be lower than in systems that structurally rely on user fee charges. Meanwhile, also in line with prior empirical analyses on household health expenditure [46,47], our income elasticity, estimated based on the SES coefficient in the two-part model, was inelastic or near to zero. This implies that a 1% rise in income will lead to far less than a 1% increase in absolute OOP expenditure, also indicating the less prominent role of SES in directly shaping absolute OOP expenditure on CNCDs in Malawi. Still, the poorer quartile spent a considerably higher proportion of their monthly per capita household expenditure on healthcare needs related to CNCDs than wealthier quartiles. This finding is consistent with prior studies in Malawi [50] and in other LMICs [6,19,30], indicating that OOP expenditure on healthcare is generally regressive. This finding is particularly worrisome since the negative effect of regressive payments for health has been widely documented in the literature [10].

In our analysis, use of formal care was negatively associated with the probability of incurring, but positively associated with the magnitude of the incurred OOP expenditure. Given that the vast majority of those using formal services actually use public facilities (more than 90% in our sample), this finding can most likely be explained by the fact that public facilities are expected to provide care free of charge at point of use. Still, previous studies have repeatedly indicated that public facilities fall short of providing all the services formally included in the EHP. Thus, it is plausible to assume that if in need of special care (including specific diagnostics and drugs), people would have incurred higher expenditure due to the need to purchase privately services not always available at the public facilities [24,25,54–56]. The plausibility of this finding is well aligned with the evidence emerging from a parallel qualitative study which indicates important gaps in service coverage and financial protection, inducing rural Malawians to seek care privately to compensate for weaknesses in the public system [55]. In principle, those who choose to use public facilities should be able to access essential care, including laboratory tests and drugs, free of charge and should be left to pay only for transportation costs. The fact that more than 60% of those who incurred OOP expenditure did not spend anything on transportation suggests that higher OOP expenditure among users of formal care is driven by direct medical expenditure. In turn, this suggests that tests and drugs are not always available free of charge, probably due to underfunding at the system level [25]. Further qualitative investigation is necessary to understand if higher OOP spending is incurred because patients are charged informal fees or because they are sent to private structures to purchase material which is missing at public facilities. In either case, the expectation of ultimately facing higher costs if seeking formal rather than informal care may very well act as a deterrent when making decisions on how to treat CNCDs [6], potentially leading to improper disease management and worse long-term health outcomes [4].

Our prior work already indicated that health seeking behavior differed substantially between individuals suffering from CNCDs covered by the EHP and those suffering from CNCDs not covered by such program [26]. Our current analysis on expenditure confirmed that suffering from CNCDs targeted by EHP screening programs was positively associated with a higher magnitude of OOP expenditure. This finding appeared surprising, since we expected to observe a lower OOP expenditure on CNCDs targeted by the EHP, due to the fact that an explicit aim of the EHP is to serve as a financial protection mechanism against illness-induced costs. Our opposite findings suggest that, in its current design, the EHP as currently implemented has not achieved the goal of removing the financial burden induced by selected CNCDs. A possible explanation for this surprising finding may be found by looking at the literature, which has amply documented a general inability of the system to provide services in the quantity and quality stipulated by the EHP [24,56].

The positive association detected between being a household head and the amount of OOP spending on CNCDs aligns with the vast body of evidence indicating that the intra-household allocation of resources for health in SSA prioritizes productive household members [57] and particularly the family head [19,21]. Given that we controlled for age, it is unlikely that the effect detected reflects individual CNCD risk profiles, but more likely that it reflects actual preferences on intra-household resource allocation. Similarly, consistent both with theoretical models of demand for health care [58] and with prior empirical studies from LMICs [19,30,32,46,52], we found that OOP expenditure was higher among individuals whose household head was literate. Literate household heads have better access to health information. This is likely to result in better health knowledge and, in turn, in different decisions regarding investments in health. Living in a household with a higher proportion of individuals suffering from a chronic illness was negatively associated with the magnitude of individual OOP expenditure on CNCDs. This indicates that intra-household resource allocation is more problematic in households where multiple family members suffer from one or more CNCDs [26], given that in a context of generalized poverty, limited resources have to be shared between many individuals.

Contradicting prior evidence on acute conditions [19,59], we found perceived disease severity to be positively associated with the probability of incurring OOP expenditure, but not with the magnitude of the expenditure. In line with prior studies [60–62], illness duration was instead found to be positively associated with the magnitude of OOP expenditure. These findings indicate that perceived illness severity, although relevant for an individual’s perceived quality of life, is less suitable for representing actual severity of disease than illness duration for the purpose of analyzing OOP expenditure on CNCDs. The fact that illness duration is an important predictor of OOP expenditure on CNCDs is not surprising, since CNCDs normally aggravate with time and thus require more intense medical care [4]. From the standpoint of financial protection, this obviously calls into question the capacity of the Malawian healthcare system to cater for individuals who need relatively expensive lifelong care, while still struggling to curb mortality due to infectious diseases and lack of qualified maternal care [63].

A few methodological limitations of this study need to be acknowledged. First, since our study is based on self-reported data, OOP expenditure on CNCDs might be underestimated due to recall bias [64,65]. Second, categorizing reported information on chronic illnesses and complaints does not allow for an assessment of the actual clinical correctness of underlying diagnoses or symptoms. In line with what is frequently done for studies relying on self-reported morbidity [66–68], we used an approach that allows us to transform, as accurately as possible, the information on chronic ailments perceived and reported by lay people into a commonly used CNCD categorization framework based on WHO criteria [1]. It has to be recognized that this approach does not necessarily reflect the epidemiological burden of CNCDs among the study sample, as no clinical assessments were conducted to verify the reported illness information. Details on the approach used here are presented in our previous publication on CNCD-related health seeking behavior [26]. Third, being one of the first exploratory studies on determinants of OOP expenditure on CNCDs in SSA, this study is based on a cross-sectional design. Longitudinal study designs will be more suitable for future research on the causality between the explanatory variables identified here and OOP expenditure on CNCDs. As an explorative study on health seeking behavior and OOP expenditure on CNCDs in SSA, it makes sense that we use proximal similarity model, which refers to generalizing the findings beyond the source population whose setting is more or less similar to that of the source population [69,70], to discuss generalizability. By detailed introducing our study area, other researchers can fully understand similarities and differences between our study setting and the specific context that they concern and make decisions on whether our findings can be generalized to their context. In addition, our findings are related to OOP expenditure on CNCDs. One needs to be cautious when he/she generalizes our findings to OOP expenditure for the general condition.

## Conclusions

Our study showed that even in a context where essential care for CNCDs is supposedly free of charge at point of use, OOP expenditure imposes a considerable financial burden on rural households, especially among the very poor. This indicates the existence of important gaps in service coverage and financial protection in the current government’s universal health coverage policy. Existing policy needs to translate into direct action plans to ensure that in a context of generalized poverty, households facing multiple health burdens (from acute, chronic, and maternal conditions) benefit from proper access to the services and adequate financial protection. This study describes patterns of and factors associated with OOP expenditure on CNCDs in Malawi, which provides useful information to develop action plans aimed at reducing the financial burden due to CNCDs in the setting.
